# Immigrants in the Income Elite in Germany: The Role of Immigrant‐Native Households

**DOI:** 10.1111/1468-4446.70007

**Published:** 2025-07-01

**Authors:** Florian Zimmermann, Matthias Collischon, Anja Wunder

**Affiliations:** ^1^ Research Data Centre Institut für Arbeitsmarkt‐ und Berufsforschung (IAB) Nürnberg Germany; ^2^ Research Department PASS Institut für Arbeitsmarkt‐ und Berufsforschung (IAB) Nürnberg Germany

**Keywords:** education, elite, household, immigrants, self‐employment

## Abstract

Although studying elites is a growing strand of scholarship in social sciences, the literature is mostly migration‐blind. In this research note, we examine the role of household composition for immigrants' pathways to the elite of the household income distribution in Germany. Distinguishing between native‐native, immigrant‐native, and immigrant‐immigrant households, we investigate the propensity of being in the income elite by household composition and whether education and self‐employment, two major pathways into the income elite, differ by household composition. We hypothesize that immigrants in immigrant‐native households benefit from their native partner's host‐country resources and support. Using data from the German Microcensus from 2009 to 2019 covering around three million observations, we show that immigrant‐native households have a higher propensity of belonging to the income elite compared to immigrant‐immigrant households. Surprisingly, we find no differences between immigrant‐native and native‐native households. In addition, we demonstrate that the positive association between education, self‐employment and elite membership is stronger for immigrant‐native households compared to immigrant‐immigrant households. Overall, our research note highlights the importance of the household context for immigrants' access to the income elite.

## 1 Introduction

Due to widening inequalities within societies, the study of elites has gained increasing attention in social sciences, such as Economics (e.g., Saez and Zucman 2016) and Sociology (Cousin et al. 2018; Keister and Lee 2017; Rahman Kahn 2012). Elites are considered to have substantial social, economic, and political influence, partly due to unprecedented access to political decision‐makers. This privileged social position of elites can contribute to the reproduction of social inequality in societies (Keister and Lee 2017; Laurison and Friedman 2016) and increase social stratification (Collischon et al. 2024). Despite the growing body of literature on elite research, previous research is mostly migration‐blind (Cousin et al. 2018), probably due to a lack of suitable data with sufficient observations for this subgroup. Considering that large shares of the population of Western Countries are foreign‐born—e.g., 14% of the population of the United States and 16% of the population of Germany (OECD 2024)—research neglects an important dimension of social inequality.

While general pathways of immigrants to the income elite, such as education or self‐employment, have been investigated (e.g., Keister and Lee 2017), research neglects the household point of view. Household context is important for understanding immigrant‐native inequalities (e.g., OECD 2023). In this context, previous research has shown that immigrants might benefit from cohabiting with natives because natives could help immigrants to access host‐country specific resources, such as native networks, and skills, such as language proficiency (Lancee 2012; Nottmeyer 2015; Meng and Gregory 2005). Thus, natives' support can improve immigrants' employment chances and increase their wages (Elwert and Tegunimataka 2016). Yet it remains unclear how household constellation, i.e., immigrant‐native or immigrant‐immigrant households, affects immigrants' chances of being in the income elite. Thus, our R1 is: *Do households differ in their propensity of belonging to the income elite depending on the household composition, that is, native‐native, immigrant‐native, and immigrant‐immigrant households?*

In this research note, we contribute to the literature by bridging the household literature (e.g., Smock and Schwartz 2020), migration studies (e.g., Platt et al. 2022), and the literature on elites (e.g., Keister 2014). Despite the importance of the topic, we are not aware of a single study focusing on household composition in the context of immigrants in the income elite. Thus, we contribute to the literature in two ways: First, we provide first cross‐sectional insights on the interplay between migration and the household context in the context of elite membership. Second, we investigate how common pathways to the income elite differ by household composition. Based on previous research we focus on the role of education and self‐employment. By doing so, we contribute to a deeper understanding of the role of social networks and host‐country skills in shaping social inequality.

To answer our research question, we use the 2009 to 2019 waves of the German Microcensus (DeStatis 2020), which is a representative 1% sample of German households, encompassing around three million observations. This large number of cases uniquely enables subgroup analyses of households in the income elite. As a household survey, the Microcensus allows us to include information such as education and employment status of both household members and enables analyses of immigrants in the income elite in the household context. Because the Microcensus is a stratified sample with compulsory participation, it is not affected by non‐response bias, which is especially high for immigrants (Laganà et al. 2013) and individuals in the income elite (Blanchet et al. 2022). Thus, the Microcensus offers a unique opportunity to investigate immigrants in the income elite.

### 1.1 State of Research

#### 1.1.1 Immigrants and Natives in the Income Elite

On the individual level, immigrants on average earn lower wages than natives and have a lower probability of being in the income elite (Collischon et al. 2024). Thus, we also expect households with two immigrants (immigrant‐immigrant households) to have a lower probability of being in the income elite compared to native‐native households (H1a). For immigrant‐native households, we expect a higher probability of being in the income elite compared to immigrant‐immigrant households (H1b) for two reasons: First, part of the household income is contributed by a native, who has a higher probability of being in the income elite compared to immigrants (Collischon et al. 2024). Second, immigrants in immigrant‐native partnership have higher incomes than immigrants in immigrant‐immigrant partnerships, for example due to faster integration (Elwert and Tegunimataka 2016; Meng and Gregory 2005). Since immigrants are still disadvantaged in the labor market compared to natives, even when married to a native, we expect that immigrant‐native households have a lower probability of being in the income elite compared to native‐native households (H1c).

#### 1.1.2 Returns to Education and Self‐Employment of Immigrants

In general, education and self‐employment increase the propensity of women (Yavorsky et al. 2019) and immigrants (Collischon et al. 2024) having a personal income elite status. Yet, the association between education or self‐employment and income elite status is lower for immigrants compared to natives due to a lack of host‐country specific resources, such as networks (Lancee 2012), and host‐country specific skills, such as human capital and language skills (Chiswick and Miller 2012).

Native partners can help immigrants to achieve higher returns to education. Native partners can facilitate integration, including economic integration (Meng and Gregory 2005), and might have knowledge that immigrant partners lack, such as information about host‐country rules and customs, and offer support for host‐country language acquisition (Elwert and Tegunimataka 2016). Furthermore, immigrants with native partners can gain access to native networks (Lancee 2012; Nottmeyer 2015). These networks are more efficient in providing jobs matching immigrants' qualifications (Furtado and Theodoropoulos 2010), thereby increasing the returns to education. In summary, we expect a stronger association between education and income elite status for immigrants in immigrant‐native households compared to immigrants in immigrant‐immigrant households (H2a).

Regarding the returns to self‐employment, immigrant‐native partnerships are closely linked to the acquisition of information and networks (Elwert and Tegunimataka 2016). Having a native partner may provide immigrants with know‐how on administrative, legal, and financial knowledge, and facilitate communication with financial institutions or suppliers, increasing chances of success for self‐employment (Kanas et al. 2009). Accordingly, businesses of immigrants married to natives have a lower probability of exiting entrepreneurship (Georgarakos and Tatsiramos 2009). Furthermore, immigrants with native partners have better employment alternatives (Nottmeyer 2015), leading to less adverse selection into self‐employment compared to immigrants in immigrant‐immigrant households. In summary, we expect that self‐employment has a stronger association with income elite status for immigrants in immigrant‐native households compared to immigrants in immigrant‐immigrant households (H2b).

## 2 Data

We use data from the German Microcensus from 2009 to 2019 (DeStatis 2020). The Microcensus is an annual 1% sample of German households (i.e., around 400,000 households per year) collected by the Federal Statistical Agency. The data contain information on sociodemographic characteristics as well as the household composition. Participation in the survey is mandatory, ensuring little non‐response and high data quality. Regarding migration status, our analysis compares first‐generation immigrants who do not possess a German citizenship to native Germans.

Income is surveyed for households as monthly net income from all sources in 24 categories ranging from below €150 to above €18,000 (Table A1). This measure captures income from labor and financial returns from assets or real estate. Because we investigate the household context, we focus on cohabiting heterosexual couples.^1^ These restrictions leave us with 1,050,554 households of whom 4.13% classify as elite households with an income over €7,500, a definition that was chosen in a comparable study at the household level with the same dataset (Collischon 2023).^2^ To show that this threshold is not driving our results, we use a stricter threshold of €18,000 in a robustness check, thereby classifying 0.39% as elite households. Appendix Table A2 shows on overall overview of sample means for our groups of interest.

### 2.1 Analytical Strategy

Our empirical analysis starts by descriptively investigating top‐earner household status by household type, that is native‐native, immigrant‐native, and immigrant‐immigrant households. In this descriptive analysis, we use the cross‐sectional survey weights provided by the German statistical agency to allow for the generalizability of the findings to the residential population in Germany. To the best of our knowledge, this analysis provides novel descriptives of elite households that have not been published previously.

In our main analysis, we use an unweighted regression analysis to investigate the correlates of being in an elite income household. We estimate the following linear probability model (LPM) separately by household type:

$${topearner}_{it}=\beta_{0}{+\beta }_{1}{male\_education^{\prime }}_{it}+\beta_{2}{male\_empstat^{\prime }}_{it}+\beta_{3}{female\_education^{\prime }}_{it}+\beta_{4}{female\_empstat^{\prime }}_{it}+\gamma Z_{t}^{\prime }+\delta X_{it}^{\prime }+\epsilon_{it}$$

where topearner is the top earner status of the household i in year t, which we define as having a net household income of €7500 or more per month. *Male_education* is a set of indicator variables for the male's education, and *male_empstat* is the man's employment (that also contains one category for non‐employment) status. Additionally, we control for the woman's education (*female_education*) and employment status (*female_empstat*). We also control for two sets of confounders: *Z* is a set of survey year indicators, and *X* is a set of covariates, namely age (measured as categories: below 25, 5‐year steps to 65, and above 65^3^), living in East Germany, having children and being married. This estimation allows us to disentangle the determinants of elite household status while holding potentially confounding factors constants. We use heteroskedasticity‐robust standard errors in the estimations.

## 3 Results

### 3.1 Time Trends in Belonging to the Income Elite by Household Composition

**FIGURE 1:**
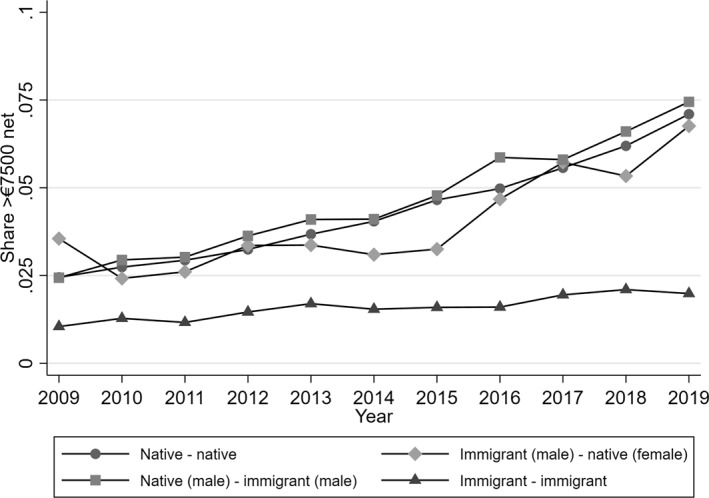
Share of households in the income elite by household composition between 2009 and 2019. *Source:* own calculations using the German Microcensus data.

We begin by investigating time trends in the probability of belonging to the income elite over the observation period by household composition (Figure 1). The share of immigrant‐immigrant households with an income over €7500 is stagnant over time, while the share of native‐native and immigrant‐native households in the income elite increases from around 3% in 2009 to approximately 8% in 2019. Thus, we can observe a widening gap between immigrant‐immigrant households and native‐native or immigrant‐native households over time.

When comparing the shares of the different household compositions in the income elite, the share of immigrant‐immigrant households is lower than the share of native‐native or immigrant‐native households in the income elite (Figure 1), supporting our hypotheses H1a and H1b. Notably, it does not seem to matter whether the male or female in the household is the immigrant in native‐immigrant households. Surprisingly, the difference between native‐native and immigrant‐native households is negligible and not statistically significant, which is evidence against H1c.

### 3.2 Regression Results

**FIGURE 2:**
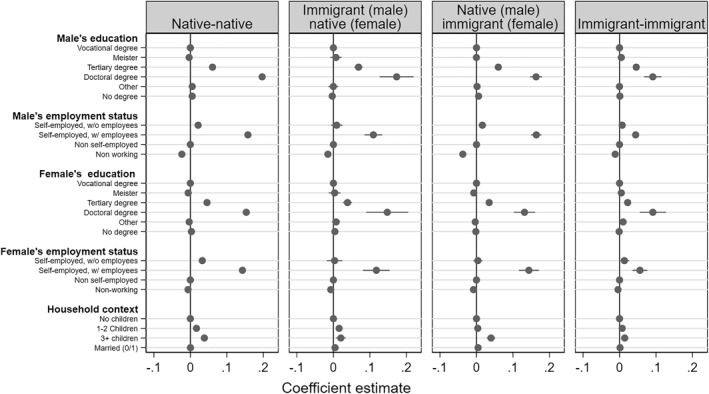
Marginal effects of selected variables on the propensity of belonging to the income elite by household composition. *Source:* own calculations using the German Microcensus data.

Figure 2 shows the coefficient estimates of the LPMs for individual and partner characteristics (Table A3 shows the coefficient estimates). Both education and self‐employment are positively associated with elite income status for all household compositions and regardless of gender. When comparing the association between education and self‐employment in immigrant‐native households with immigrant‐immigrant households, we find stronger association for immigrant‐native households independent of the immigrant's gender. These findings are evidence that immigrants in immigrant‐native households have higher returns to education (H2a) and self‐employment (H2b).

When comparing immigrant‐native to native‐native households, we surprisingly find no difference in the association between education or self‐employment and elite income status between these two household constellations. This finding is again robust to an immigrant's gender.

### 3.3 Robustness Checks

**FIGURE A1:**
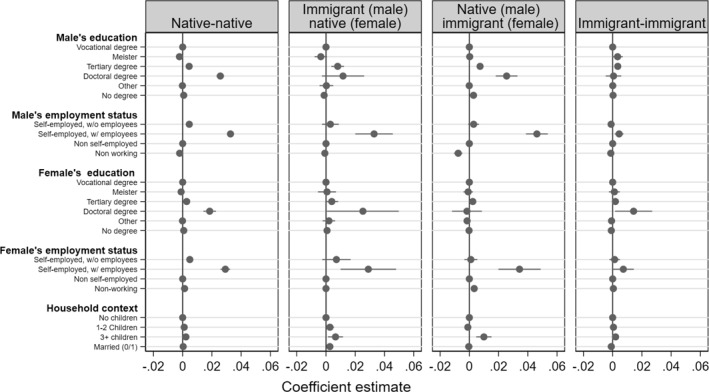
Marginal effects of selected variables on the propensity of belonging to the income elite by household composition; €18,000 cutoff. *Source:* Own calculations using the German Microcensus data.

Previous literature (e.g., Yavorsky et al. 2019) defines elite membership as the top 1% of the household income distribution. We repeat our analysis with a cutoff of monthly household income equal or larger than €18,000 per month, representing the top 0.39% of households. The qualitative results hardly changing (Figure A1), indicating the robustness of our baseline findings.

**FIGURE A2:**
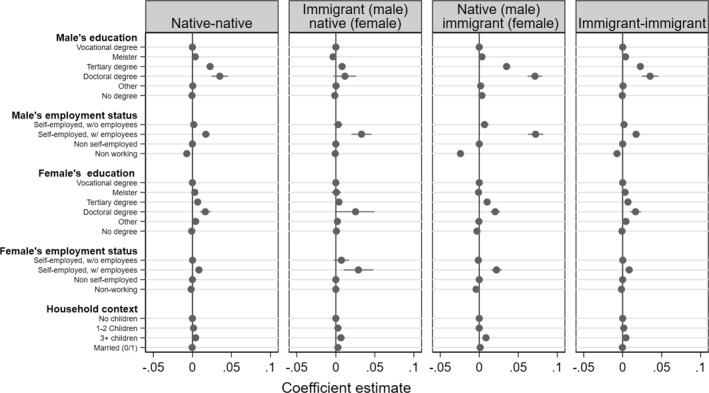
Marginal effects of selected variables on the propensity of belonging to the income elite by household composition; marginal effects from cloglog regression. *Source:* Own calculations using the German Microcensus data.

Because our outcome of interest ‐ elite membership ‐ is by definition a rare event, we use a complementary log‐log (cloglog) regression as a robustness check. Again, the results remain robust (Figure A2).

## 4 Conclusion

Elite research is increasingly becoming the focus of research in social sciences as elite status may result in higher political, cultural, and social influence (Keister and Lee 2017). Since immigrants are underrepresented by about 46% in the income elite in Germany (Collischon et al. 2024), elite membership of immigrants is an important dimension for social inequalities. While research has investigated immigrants' pathways into the income elite (Collischon et al. 2024), the household context's role remained unclear. In this note, we examined the role of the household constellation in relation to first‐generation immigrants' elite membership. Using data from the German Microcensus from 2009 to 2019, we investigated native‐native, immigrant‐native and immigrant‐immigrant households' pathways in the income elite to contribute to a deeper understanding host‐country resources' role in the context of income elites.

Our results show that immigrant‐immigrant households are significantly less likely to be in the income elite compared to immigrant‐native and native‐native households. Surprisingly, immigrant‐native and native‐native households do not differ in terms of their probability of belonging to the income elite. Digging deeper into the pathways to the income elite, our results show that tertiary education and self‐employment are positively associated with household income elite status, which is similar to previous research (Collischon 2023; Yavorsky et al. 2019). Yet, the strength of the association between education or self‐employment and elite status differs by household constellation. For immigrants in immigrant‐native households, the association is stronger than for immigrants in immigrant‐immigrant households, potentially due to the access to host‐country specific resources and skills. Thus, our study deepens our understanding of social inequality by highlighting the role of the household constellation for immigrants' pathways to the income elite.

A major limitation of our research note is that we cannot investigate whether and to what extend selectivity in partnerships influences our results. For example, if immigrants that are already integrated and have higher language skills are also more often in partnerships with natives (Lichter et al. 2015; Georgarakos and Tatsiramos 2009), we could overestimate the effect of immigrant‐native cohabitation on pathways to the income elite. Since Elwert and Tegunimataka (2016) show that the wage premium of immigrants in immigrant‐native relationships comes directly after relationship formation, we assume that cohabitation also at least partially causes the higher probability of being in the income elite, albeit we cannot directly investigate potential selectivity.

Overall, our research note highlights the importance of the household context for immigrants' access to the income elite and lays the groundwork for future research regarding the interplay between household constellation and elite status. For example, future research could focus on identifying relationship dynamics and specific mechanisms within partnerships. Furthermore, disentangling the role of underlying mechanisms, such as native social networks and language skills, could show the relative strength of these mechanisms.

## Data Availability

The data that support the findings of this study are available from DeStatis Forschungsdatenzentrum. Restrictions apply to the availability of these data, which were used under license for this study. Data are available from https://www.forschungsdatenzentrum.de/de/haushalte/mikrozensus with the permission of DeStatis Forschungsdatenzentrum.

## Endnotes

^1^
Homosexual couples make up less than 0.2% of our overall sample. We exclude those, because they may differ in their household dynamics from heterosexual couples. Unfortunately, we cannot investigate this group more in detail due to a low number of cases.

^2^
Note that including single households, households with an income over €7500 would encompass 2.85% of households. The number is mechanically lower compared to our sample, as we exclude all single‐person households with this definition.

## Appendix

**TABLE A1:** Income brackets and distribution in the data (weighted).

Household income bracket in Euros	Share in percent
< 150	0.01
150–299	0.02
300–499	0.04
500–699	0.20
700–899	0.57
900–1099	1.32
1100–1299	2.46
1300–1499	3.69
1500–1699	4.85
1700–1999	8.21
2000–2299	9.10
2300–2599	9.02
2600–2899	8.17
2900–3199	7.75
3200–3599	9.05
3600–3999	7.29
4000–4499	7.35
4500–4999	5.41
5000–5499	4.04
5500–5999	2.87
6000–7499	4.51
7500–9999	2.45
10,000–17,999	1.21
18,000+	0.39

*Source:* Own calculations using the German Microcensus data.

**TABLE A2:** Sample descriptives (weighted).

	(1)	(2)	(3)	(4)	(5)	(6)
		Income <= 7500€			Income >€ 7500	
	Native‐native	Immigrant‐Native/Native‐immigrant	Immigrant‐immigrant	Native‐native	Immigrant‐Native/Native‐immigrant	Immigrant‐immigrant
Male's age	56.87	57.90	48.72	53.84	53.24	49.27
Male's education						
Education: Vocational degree	0.59	0.50	0.31	0.21	0.17	0.13
Education: Meister	0.10	0.08	0.02	0.07	0.05	0.02
Education: Tertiary degree	0.19	0.22	0.14	0.52	0.55	0.57
Education: Doctoral degree	0.02	0.03	0.01	0.15	0.18	0.11
Education: Other	0.04	0.04	0.04	0.02	0.02	0.02
Education: No degree	0.06	0.13	0.49	0.02	0.04	0.15
Education partner: Vocational degree	0.59	0.50	0.31	0.21	0.17	0.13
Male's employment status						
Self‐employed, w/o employees	0.04	0.05	0.04	0.07	0.10	0.09
Self‐employed, w/employees	0.04	0.04	0.04	0.29	0.27	0.16
Non self‐employed	0.53	0.46	0.58	0.54	0.53	0.68
Non working	0.39	0.45	0.34	0.09	0.11	0.08
Female's education						
Education partner: Meister	0.58	0.42	0.21	0.36	0.24	0.14
Education partner: Tertiary degree	0.04	0.03	0.01	0.04	0.03	0.02
Education partner: Doctoral degree	0.12	0.18	0.14	0.40	0.49	0.50
Education partner: Other	0.01	0.01	0.01	0.07	0.08	0.07
Education partner: No degree	0.09	0.08	0.04	0.08	0.06	0.06
Female's employment status						
Employment partner: Self‐employed, w/o employees	0.02	0.03	0.02	0.07	0.08	0.06
Employment partner: Self‐employed, w/employees	0.01	0.01	0.01	0.09	0.09	0.06
Employment partner: Non self‐employed	0.52	0.40	0.40	0.63	0.56	0.51
Employment partner: Non working	0.45	0.55	0.57	0.21	0.28	0.37
Household context						
Married (0/1)	0.93	0.94	0.98	0.97	0.96	0.98
Child in HH (0/1)	0.37	0.36	0.61	0.58	0.57	0.70
Number children	0.65	0.62	1.27	1.12	1.07	1.43
East Germany (0/1)	0.25	0.24	0.22	0.22	0.21	0.21
Observations	863,109	79,030	65,046	38,622	3683	1061

*Source:* Own calculations using the German Microcensus data.

**TABLE A3:** Regression results corresponding to Figure 2.

	(1)	(2)	(3)	(4)
	Native‐native	Immigrant (male)—native (female)	Native (male)—Immigrant (female)	Immigrant‐immigrant
Male's education				
Vocational degree	Ref.			
Meister	−0.003^***^	0.007	−0.000	0.005
	(0.001)	(0.008)	(0.002)	(0.004)
Tertiary degree	0.061^***^	0.069^***^	0.060^***^	0.046^***^
	(0.001)	(0.006)	(0.002)	(0.003)
Doctoral degree	0.197^***^	0.173^***^	0.163^***^	0.091^***^
	(0.003)	(0.024)	(0.009)	(0.012)
Other	0.005^***^	−0.000	0.002	−0.000
	(0.001)	(0.007)	(0.003)	(0.002)
No degree	0.005^***^	−0.003	0.006^**^	0.001
	(0.001)	(0.002)	(0.002)	(0.001)
Male's employment status				
Self‐employed, w/o employees	0.021^***^	0.009	0.016^**^	0.007^*^
	(0.001)	(0.008)	(0.005)	(0.003)
Self‐employed, w/employees	0.158^***^	0.110^***^	0.164^***^	0.044^***^
	(0.002)	(0.012)	(0.007)	(0.005)
Non self‐employed	Ref.			
Non working	−0.023^***^	−0.015^***^	−0.037^***^	−0.012^***^
	(0.001)	(0.004)	(0.003)	(0.001)
Female's education				
Vocational degree	Ref.			
Meister	−0.006^***^	0.003	−0.007	0.005
	(0.001)	(0.008)	(0.004)	(0.005)
Tertiary degree	0.046^***^	0.039^***^	0.035^***^	0.022^***^
	(0.001)	(0.006)	(0.003)	(0.003)
Doctoral degree	0.153^***^	0.148^***^	0.132^***^	0.091^***^
	(0.005)	(0.029)	(0.015)	(0.018)
Other	−0.003^***^	0.007	−0.003	0.010^**^
	(0.001)	(0.006)	(0.002)	(0.003)
No degree	0.003^***^	0.004	−0.002	−0.001
	(0.000)	(0.003)	(0.001)	(0.001)
Female's employment status				
Self‐employed, w/o employees	0.033^***^	0.003	0.004	0.013^*^
	(0.002)	(0.011)	(0.006)	(0.007)
Self‐employed, w/employees	0.143^***^	0.118^***^	0.144^***^	0.056^***^
	(0.003)	(0.019)	(0.014)	(0.010)
Non self‐employed	Ref.			
Non‐working	−0.006^***^	−0.008^***^	−0.007^*^	−0.004^***^
	(0.001)	(0.002)	(0.003)	(0.001)
Household context				
Married (0/1)	0.000	0.005	0.004	0.001
	(0.001)	(0.005)	(0.003)	(0.003)
No children	Ref.			
1‐2 children	0.017^***^	0.016^***^	0.004	0.007^***^
	(0.001)	(0.004)	(0.003)	(0.001)
3+ children	0.038^***^	0.020^**^	0.040^***^	0.014^***^
	(0.001)	(0.007)	(0.006)	(0.002)
East Germany (0/1)	−0.007^***^	−0.006	−0.003	−0.002
	(0.000)	(0.004)	(0.002)	(0.001)
Constant	−0.014^***^	−0.015^*^	−0.010^***^	−0.010^**^
	(0.001)	(0.007)	(0.003)	(0.003)
Observations	901,734	15,298	67,415	66,107

^*^ *p* < 0.05.

^**^ *p* < 0.01.

^***^ *p* < 0.001.
